# Spirituality and mental health – investigating the association between spiritual attitudes and psychosomatic treatment outcomes

**DOI:** 10.3389/fpsyt.2024.1497630

**Published:** 2025-01-27

**Authors:** Thilo Hinterberger, Nike Walter

**Affiliations:** Research Section of Applied Consciousness Sciences, Department of Psychosomatic Medicine, University Hospital Regensburg, Regensburg, Germany

**Keywords:** spirituality, mental health, psychosomatic medicine, questionnaire, treatment outcome

## Abstract

**Background:**

The relationship between spirituality and mental health has garnered attention, fostering overall well-being. Spirituality, posited as a protective factor, may enhance resilience and provide meaning, thus benefiting mental health. This study aims to identify spirituality-associated factors influencing clinical outcomes in psychosomatic inpatients and validate the Transpersonal Spirituality Inventory (TSI).

**Methods:**

The study involved 4952 psychosomatic inpatients completing the Transpersonales Vertrauen (TPV) and 7739 patients completing the TSI, with assessments conducted at admission and discharge. Additional instruments included the ISR for symptom rating and the LK-18 for life skills. Factor analysis and Spearman’s rank correlation were used to evaluate the validity of TSI and the relationship between spirituality and clinical outcomes.

**Results:**

Factor analysis confirmed the TSI’s two-factor structure: “centered connectedness” (F1) and “transcendent orientation” (F2), with satisfactory internal consistency (Cronbach’s α = 0.824 for F1 and 0.644 for F2). Higher spirituality levels, particularly in transpersonal trust and centered connectedness, correlated with lower depression and psychosomatic symptoms (ISR). Although these correlations were generally weak, significant associations were found between spirituality and life competences, particularly in meaning, belief, and values (r = .595 for TPV and .598 for TSI F1).

**Conclusion:**

Despite correlations between psychosomatic health and spirituality as measured with TPV and TSI were rather small several spirituality items could be identified which seem to have a prominent connection to the diagnosis and development of psychosomatic health. Specific spiritual attitudes, such as access to inner stillness and connection to a greater whole, were linked to positive treatment responses and reduced symptom burden. These findings suggest potential benefits in incorporating spirituality into psychosomatic treatments, though its complex and multifaceted nature warrants further investigation.

## Introduction

**Figure 1:**
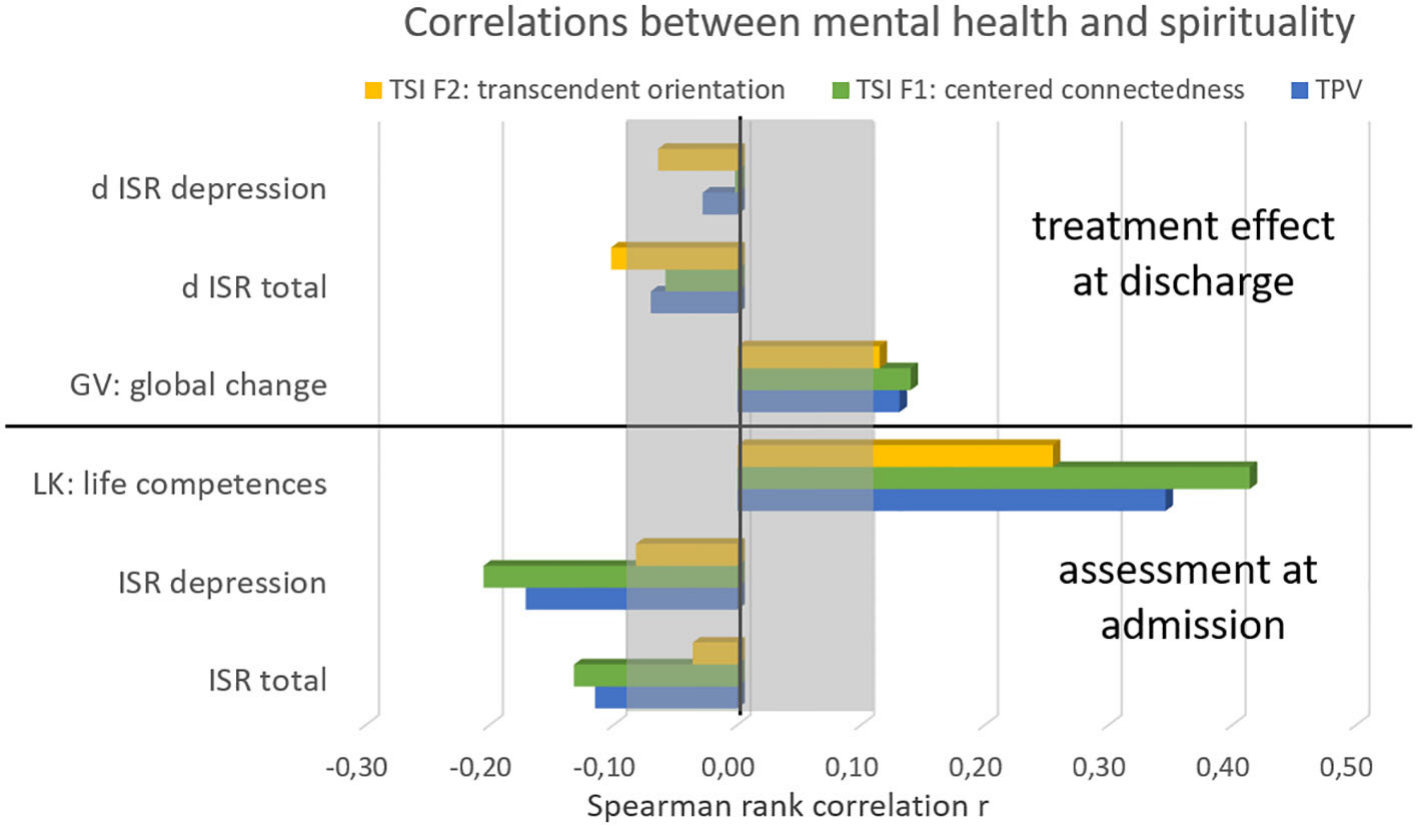
Correlations of spirituality scores with mental health at admission and with treatment effects. The lower part refers to the ISR before clinical treatment, the lower part shows the correlations to the treatment effects. d ISR denominates the difference of ISR scores between admission and discharge.

The relationship between spirituality and mental health has become an increasingly intriguing topic within the realms of psychology and psychiatry (1, 2). Numerous studies have demonstrated the positive influence of spirituality on both physical and mental health, as well as its association with other favorable outcomes like subjective well-being, health-related quality of life, coping abilities, recovery from mental illnesses, and reduced addictive or suicidal behaviors (3–5) Klicken oder tippen Sie hier, um Text einzugeben. Additionally, spiritual practices such as meditation and prayer have shown positive impacts on mental health by reducing stress and promoting relaxation. Moreover, these practices can aid in enhancing emotional regulation, increasing self-awareness, and fostering inner peace (6–8). Given these findings, it appears beneficial to develop interventions that incorporate spirituality into psychosomatic treatments.

However, it is essential to acknowledge that spirituality is a multidimensional theoretical construct, making its scientific evaluation a complex task (9). Furthermore, the relationship between spirituality and mental health is bidirectional and intricate, with the presence of potential negative effects as well (10).

The primary objective of this study is to identify aspects and factors of spirituality associated with psychosomatic symptoms, mental health, and life competences which also may influence clinical outcomes in a sample of psychosomatic inpatients and further to validate a short questionnaire for clinical use, the Transpersonal Spirituality Inventory. Despite the growing body of research in this area, there remains a research gap in understanding the precise key aspects through which spirituality impacts mental health. This study is based on the assumption that spirituality is a broad term including many facets which in total only show a minor relationship to mental health. However, by investigating these factors in detail on a single-item basis valuable insights can be derived to inform the development of targeted and effective interventions for psychosomatic patients that consider the role of spirituality in their well-being and recovery.

## Methods

### Outcome measures

TPV inventory: The TPV, which stands for “Transpersonales Vertrauen” in German, was developed by Bantelmann and Belschner to evaluate transpersonal confidence, faith, and beliefs. It is a component of a larger inventory known as the FIG-50 (Questionnaire Integral Health - “Fragebogen Integrale Gesundheit 50”). The TPV consists of 10 questions that gauge an individual’s spirituality using a 4-point scale leading to a single factor calculated as the summarized total score (11). Its validity has been confirmed, and it demonstrates strong internal consistency with a Cronbach’s alpha of 0.92 (12).

TSI inventory: The TSI (Transpersonal Spirituality Inventory) is a short six item inventory designed for clinical screening assessing two aspects of spiritual attitudes: centered connectedness (F1) and transcendent orientation (F2). Each of them includes three items on a 4-point Likert-scale. The construct validity was calculated within this study group and Cronbach alpha of .82 for F1 and .64 for F2 (see result section).

ISR inventory: The ISR is an inventory for psychosomatic symptom rating based on the ICD-10 classification system. It serves as a license-free alternative to the well-known international SCL90 (Symptom Checklist 90) [49]. In a comparative study, the total scales of ISR and SCL-90 showed a strong correlation of r=0.833. However, the intercorrelations within the ISR (r=0.32) were lower compared to those within the SCL-90 (r=0.65) (13). The ISR comprises 29 items, and respondents rate each item on a 5-point scale. The inventory assesses various factors, including depression, anxiety, obsession, somatic symptoms, eating disorders, as well as additional supplementary factors related to suicide, sleep problems, memory issues, sexuality, and traumatic experiences (14). The overall internal consistency of the ISR is high, with a Cronbach’s alpha of 0.92 for the total score, and the syndrome scales show good internal consistency as well, ranging between 0.78 and 0.86 (15). The changes in ISR scores were calculated by determining the difference in scores between the patients’ arrival and their release from treatment.

Life skills inventory: The questionnaire LK-18 comprises 18 items divided into 6 factors with 3 items each. These include the dimensions of positive feelings, personal competencies, engagement, meaning, success, and goal achievement as well as social relationships. Cronbach’s α values lie between .74 and .85 and for the total score .91 (16).

GV inventory: The GV, derived from the German term ‘Gesamtveränderung’ (meaning overall change), is a tool used at discharge from the clinic to evaluate global shifts in both mental and physical states. It comprises 11 questions that address various aspects of somatic and mental improvement, as well as coping abilities. The scale offers 7 response options, defined as follows: -2 = ‘essential deterioration’, -1 = ‘some deterioration’, 0 = ‘unchanged’, 1 = ‘some improvement’, 2 = ‘essential improvement’, 3 = ‘very much improvement’, and ‘was not my problem’. Consequently, a result value of 1.20 indicates that patients experienced some improvement in the respective area being assessed. Each item is examined separately, and statistical comparisons are made among different clinics. The questions in the GV inquire about enhancements in physical health, psychological well-being, self-esteem, social functioning, private relationships, occupational capabilities, motivation, understanding of the disease, future outlook, overall well-being, and daily life requirements. Apart from reporting a total score, specific constructs are also reported, which are as follows: (i) Physical health: This addresses changes in the individual’s physical well-being (referring to item 1). (ii) Mental health: This encompasses psychological health and emotional well-being (referring to items 2 and 10). (iii) Self-esteem: This relates to an individual’s perception of self-worth and confidence (referring to item 3). (iv) Coping: This construct includes various aspects such as social relationships, occupational aspects, motivation, comprehension of the disease, future orientation, and meeting daily life requirements. It also incorporates the ability to lead a self-directed life, which is explicitly encouraged and taught in the clinic setting. As a result, the GV allows for the assessment of important components of the Sense of Coherence (SOC) model and provides an extended perspective on salutogenesis. Both questionnaires GV and ISR are widely utilized across clinics by the Institute of Quality Development in Psychotherapy and Psychosomatics (IQP) as part of the foundational documentation known as Psy-BaDo (17).

### Patient population

For the TPV dataset, a total of 8396 psychosomatic inpatients were selected from the years 2014 to 2016. From the initial group, 4952 patients completed the TPV without any missing values at both the beginning and end of their clinical stay. All patients in both datasets were over 18 years old, with an average age of 46.9 years (+/- 11.1 years). The majority of patients were female with 73.1%.

Similarly, for the TSI dataset, 14712 patients received the TSI questionnaire. From this subset, 7739 patients completed the TSI without any missing values at the start and end of their clinical stay from the years 2017 to 2021. The average age was 47.5 years (+/- 12.3 years) with 70.6% female patients.

**Table 1** presents an overview including the major depression diagnosis and results of the ISR self-assessment scale.

**Table 1:** Sociodemographic and diagnostic data of the two patient populations.

	TPV	TSI
Number of patients included	4952	7739
Sex (% female)	73.1	70.6
Age (years)	46.9 ± 11.1	47.5 ± 12.3
Depression diagnosed (% with ICD10 F32/F33)	77.1	81.5
Patients with medium to severe symptom load in ISR	TPV/%	TSI/%
Total symptom load in ISR self-rating	79.3	79.0
Depression	75.6	74.7
Anxiety disorder	50.7	48.9
Compulsive disorder	34.7	33.8
Somatisation disorder	28.9	26.5
Eating disorder	21.7	19.0

### Statistical analysis

Matlab Version R2020a (MathWorks, Natrick, USA) was used for data analysis. The inclusion criteria for the analysis were twofold: firstly, patients must have completed either the TPV or TSI questionnaires in full. Secondly, patients were allowed a maximum of two missing values in the ISR, GV, and life skills questionnaires. To handle missing values, a moving median method was used for imputation in both datasets. Data sets with 3 or more missing values were excluded. As there were still enough patients included this method preserved the statistical power. Pre and post participants were identical. Notably, only 1090 patients in the TPV dataset completed the life skills questionnaire, as it was introduced later in 2016. To calculate correlations between the questionnaire items and clinical outcomes, Spearman’s rank correlation was applied after determining that the distribution was not appropriate for parametric testing by the Shapiro–Wilk test. Significance was set at *p* < 0.05.

## Results

### TSI factorial analysis

For determining the validity of the construct of the 6-item TSI questionnaire a factor analysis was carried out using the data set of 7739 patients assessed at admission in the clinic. The KMO criterion was .857 and therefore sufficient for a factorial analysis. The scree-plot suggested a two-factorial solution. The factor analysis used a varimax rotation and resulted in factor loadings according to **Table 2** and Cronbach’s alpha of.824 for F1 and.644 for F2. Factor 1 was named “centered connectedness (F1)” and the second factor “transcendent orientation (F2)”. The first two principal components explained 69.9% of the variance. An overall one-factorial solution would result in a Cronbach’s alpha of .836 with all factor loadings >.46.

### General correlations

The correlations between spirituality measures (TPV, TSI F1, TSI F2) and ISR scores indicate that higher levels of spirituality, particularly those related to transpersonal trust and TSI Factor 1, are associated with lower levels of depression and psychosomatic symptom load as measured by the ISR. However, these associations are generally weak. Among the correlations, the strongest associations were observed between spirituality measures and life competences, and meaning, belief, values with correlations ranging from .346 to .595 (**Table 3**). Specifically, there is a positive correlation between spirituality measures, particularly TPV and TSI F1 (centered connectedness), and improvements in life skills. However, the negative correlation with ISR treatment changes were weak (**Table 4**).

**Table 2:** Factor loadings of TSI items using factor analysis with varimax rotation.

Item statement	F	F1	F2	overall
1. There is a place of deep silence and confidence within me, which I have access to.	1	**.728**	.307	.727
2. I feel connected to a higher reality that instills trust in life.	1	**.638**	.540	.849
3. Spiritual practices (such as praying, chanting, spiritual songs, meditating) help me.	1	**.646**	.385	.737
4. I know that there is a greater wisdom beyond rational thinking.	2	.349	**.754**	.733
5. I feel great reverence for all living beings.	2	.302	**.362**	.469
6. I am on a quest for a greater wisdom to understand what lies behind visible manifestations.	2	.274	**.539**	.562

Bold: highest values forming factors F1 and F2.

**Table 3:** Spearman’s rank correlations between spirituality measures and ISR measures assessed at admission.

	TPV			TSI F1			TSI F2		
Spearman rank correlation r	r	CI lower	CI upper	r	CI lower	CI upper	r	CI lower	CI upper
**ISR total score**	-0,115	-0,138	*-0,092*	-0,132	-0,151	-0,114	*-0,036*	*-0,055*	*-0,017*
ISR depression	-0,171	-0,193	-0,148	-0,205	-0,223	-0,187	-0,082	-0,101	*-0,063*
ISR anxiety	*-0,054*	*-0,078*	*-0,031*	*-0,068*	*-0,087*	*-0,050*	*-0,019*	*-0,038*	*-0,001*
ISR compulsion	*-0,058*	*-0,082*	*-0,035*	*-0,078*	*-0,096*	*-0,059*	*0,002*	*-0,017*	*0,020*
ISR somatisation	*-0,001*	*-0,024*	*0,023*	*0,003*	*-0,016*	*0,022*	*0,017*	*-0,002*	*0,036*
ISR eating disorder	*-0,066*	*-0,089*	*-0,042*	*-0,049*	*-0,068*	*-0,031*	*-0,035*	*-0,054*	*-0,016*
ISR additional disorders	-0,080	-0,103	-0,057	-0,102	-0,120	*-0,083*	*-0,015*	*-0,034*	*0,003*
**LK total life skills**	**0,346**	**0,304**	**0,386**	**0,414**	**0,399**	**0,430**	0,255	0,238	0,273
LK positive emotions	0,198	0,153	0,242	0,263	0,246	0,281	0,147	0,128	0,165
LK personal competences	0,267	0,223	**0,309**	**0,376**	**0,360**	**0,392**	0,221	0,203	0,239
LK engagement	0,200	0,155	0,244	0,248	0,231	0,266	0,156	0,138	0,174
LK meaning, belief, values	**0,595**	**0,565**	**0,624**	**0,598**	**0,585**	**0,609**	**0,432**	**0,417**	**0,447**
LK success	0,170	0,124	0,214	0,239	0,222	0,257	0,126	0,108	0,144
LK social relationships	*0,098*	*0,052*	0,144	0,153	0,135	0,172	*0,081*	*0,063*	0,100

Italics: correlations <.1, Bold: moderate and strong correlations >.3, N=7739 patients at admission.

Due to the large sample size all correlations greater than .05 are significant with p<.01. **Figure 1** illustrates the correlations between spirituality measures and mental health according **Tables 3** and **4**. While the factor transcendent orientation (F2) shows almost no correlation to psychosomatic symptom loads and treatment effects, the TPV and especially centered connectedness (F1) showed some very small correlations between .10 and .20 to the total symptom load and depression score. Stronger correlations between .25 and .42 can be seen between spirituality and life skills. In contrast treatment effects seem to be hardly dependent on spiritual predispositions.

### Single-item correlations

Several noteworthy correlations were observed between the various spiritual statements, psychosomatic symptoms, and life skills (**Table 5**). Among the TSI spiritual statements, the item “There is a place of deeper silence and confidence within me that I can access” and “I am part of a greater whole, in which I am secure” showed the highest positive correlations with total life skills (r = 0.419 and r = 0.414, respectively). On the other hand, the same statements displayed weak negative correlations with the ISR total score and depression ratings (r = -0.168 and r = -0.242 for the first statement, and r = -0.132 and r = -0.205 for the second statement, respectively). Additionally, the spiritual statement related to “centered connectedness” also exhibited a strong positive correlation with total life skills (r = 0.412), and negative correlations with the ISR total score (r = -0.205) and depression ratings (r = -0.247). Other significant correlations were observed with spiritual statements such as feeling connected to a higher reality (ranging from r = 0.381 to r = 0.290), experiencing oneness with the world and cosmos (r = 0.310), and trusting in higher beings or God during difficult times (ranging from r = 0.290 to r = 0.255). Similarly, spiritual practices like praying, reciting mantras, singing spiritual songs, and meditating were also positively correlated with total life skills and negatively correlated with ISR total score and depression ratings (**Table 5**).

**Table 4:** Spirituality measures at admission correlated with treatment changes for three measures, life competences, ISR total score and ISR depression.

Spearman rank correlation r	spirituality measure		
treatment changes in	TPV	TSI F1	TSI F2
GV	.131	.140	.115
ISR total (pre-post)	*-.070*	*-.058*	-.102
ISR depression (pre-post)	*-.028*	*-.002*	*-.064*

All correlations >.05 were significant with p<.01.

**Table 5:** Spiritual statements and items from TPV and TSI correlated with life competences and ISR total and depression ratings.

Correlation TSI/TPV at admission	Life skills	ISR total	ISR Depression
There is a place of deep silence and confidence within me that I have access to	0,419	-0,168	-0,242
F1: Centered Connectedness	0,414	-0,132	-0,205
I am part of a greater whole in which I am secure	0,412	-0,205	-0,247
I feel connected to a higher reality that gives me trust in life	0,381	-0,121	-0,177
‘TPV total	0,346	-0,115	-0,171
I feel connected to a higher reality/higher being/God. I can rely on this even in difficult times	0,322	*-0,090*	-0,139
I am a human with body and intellect. And I am also inseparably connected to the cosmos.	0,310	-0,170	-0,196
Spiritual practices (e.g., praying, chanting, spiritual songs, meditating) help me	0,290	*-0,069*	-0,125
We humans cannot determine everything. There is a higher reality/higher being/God to whom I can entrust myself.	0,284	*-0,083*	-0,123
Sometimes I have the impression that I am being guided in my life by a higher insight.	0,273	*-0,058*	-0,122
Religious practices (e.g., praying, chanting mantras, singing spiritual songs, meditating) help me in difficult situations	0,272	*-0,074*	-0,135
I know that there is a greater wisdom beyond rational thinking.	0,268	*-0,069*	-0,104
I have already experienced feeling one with the world and the cosmos’	0,265	*-0,095*	-0,143
I try to entrust myself to the hand of God/a higher being	0,258	*-0,038*	*-0,093*
F2: Transcendent Orientation	0,255	*-0,036*	*-0,082*
I feel great reverence for all living things	0,232	*-0,067*	*-0,069*
I consider myself religious even if Ido not belong to any religious community	0,207	*-0,041*	*-0,084*
My soul continues to live after my death	0,183	*-0,058*	*-0,082*
I am searching for a greater wisdom to understand what lies behind visible phenomena.	0,114	*0,040*	*-0,022*

For TSI: all r values >.03 are significant with p<.05. r>.05: p<.01. For TPV: all r values >.05 are significant with p<.05. r>.08: p<.01. Items shown in yellow belong to factor 1 of the TSI, items highlighted in blue belong to factor 2 of the TSI, and items shown in green are part of the TSV. All measures are taken from the assessment at admission before treatment.

## Discussion

The study investigated the relationship between spirituality measures, psychosomatic symptoms, and life skills among psychosomatic inpatients. The primary objective was to identify factors associated with spirituality that may influence clinical outcomes. To achieve this, we validated a questionnaire, the Transpersonal Spiritual Inventory (TSI), which yielded satisfactory results in terms of construct validity and internal consistency. The factorial analysis of the 6-item TSI questionnaire indicated a two-factorial solution, with Factor 1 named “centered connectedness” (F1) and Factor 2 named “transcendent orientation” (F2). These factors showed satisfactory internal consistency, as indicated by Cronbach’s alpha coefficients. Notably, Factor 1 demonstrated stronger factor loadings and higher internal consistency compared to Factor 2.

The TSI offers a cohesive framework that captures the interplay between personal stability and broader existential connections, which is particularly relevant for patients with psychosomatic conditions. In addition, the TSI uses a non-tautological approach, as discussed in the work of Koenig, 2008 for a clear differentiation between aspects of positive mental health and spirituality (18, 19). Such approach has been deemed as essential for the development of a valid instrument (20). However, one should keep in mind that the complex and individualized nature of spirituality means that a single instrument may not be sufficient to comprehensively assess all aspects pertinent to health outcomes. For instance, awe and gratitude, highlighted as perceptive aspects of spirituality, are particularly relevant, which are not explicitly captured by the items (21). Büssing et al. (2014) demonstrated that such attitudes help patients transcend the immediate focus on illness, fostering emotional well-being and life satisfaction (22). Moreover, gratitude and awe were found to serve as coping resources, as evidenced by Vandeventer (2024), who identified a significant inverse relationship between gratitude and depressive symptoms among psychiatric inpatients (23). Thus, the TSI may complement the existing measures (24–26).

In terms of general correlations, higher levels of spirituality, particularly those related to transpersonal trust and centered connectedness (TSI Factor 1), were associated with lower levels of depression and psychosomatic symptom load as measured by the ISR. However, these associations were generally very weak and thus, might be negligeable. This suggests that while spirituality may play a role in influencing treatment outcomes among psychosomatic patients, its impact may be constrained or mediated by other factors. While spirituality may contribute to coping mechanisms and provide a sense of meaning and purpose for some individuals, its effects may be overshadowed by the multifactorial nature of other determinants such as financial situations, marital status, and education (27). For instance, individuals with strong social support networks may rely less on spiritual beliefs for coping, while those with a genetic predisposition to mental health disorders may experience symptoms regardless of their level of spirituality (28).

Further analysis of single-item correlations highlighted specific spiritual statements and practices that exhibited noteworthy associations with psychosomatic symptoms. Certain health supportive attitudes, such as having access to an inner place of deep stillness and confidence, feeling safe as part of a larger whole, being connected to a higher reality, trusting in life, and experiencing unity, were significantly correlated with positive treatment outcomes. These attitudes were associated with greater general treatment improvement, reduced ISR symptom levels, and decreased levels of depression. In contrast, a number of spiritual statements could be identified which seems not to play a role in psychosomatic health. These are mainly religious beliefs and items which were found in the TSI construct F2 termed “transcendent orientation”. These findings indicate that the implementation of spirituality-based programs in clinics or foster spirituality within the clinical setting should be beneficial as it could provide patients with coping mechanisms, existential meaning, and a sense of purpose. Therefore, the supportive statements mentioned above provide some guidance for developing such programs. In this way, spiritual interventions, such as mindfulness practices and existential therapy, could complement traditional treatment approaches by addressing the holistic needs of patients, not only in the field of psychosomatic medicine (29, 30).

The importance of integrating spirituality into clinical practice is increasingly recognized, as outlined by the DGPPN (German society for psychiatry, psychotherapy, and psychosomatic medicine) position paper on Religiosity and Spirituality in Psychiatry and Psychotherapy. The DGPPN advocates for patient-centered approaches that prioritize spiritual competence (SpK), structured assessments, and inclusion of spiritual dimensions in therapeutic plans (31). These principles align with our findings, suggesting that spiritual attitudes, such as “trusting in life” and “feeling connected to a larger whole,” correlate with improved psychosomatic outcomes. However, the DGPPN also acknowledges a persistent lack of spiritual competence among healthcare professionals as a significant barrier to implementing Spiritual Care in psychiatry and psychotherapy. Frick et al. (2020) identified that psychiatrists, therapists, and nursing staff often perceive themselves as insufficiently equipped to address patients’ spiritual needs. While older, more spiritually experienced professionals reported higher self-perceived competence, the study emphasized the need for targeted Spiritual Care training programs, particularly focusing on “self-awareness and proactive openness,” “team spirit,” and “perceptive/documentation competencies” (32). Building on these perspectives, critical insights were offered through the NERSH International Collaboration on Values in Medicine, which collected data from over 6,000 health professionals across 12 countries and six continents. Korup et al. found that 83% of physicians and 94% of nurses believed that religiosity and spirituality influenced patients’ health. Despite this high acknowledgment, formal religiosity and spirituality training remained low, with only 16% of physicians and 23% of nurses having undergone relevant education (33).

Moreover, this dataset revealed significant cross-national differences in how healthcare providers’ religious and moral values influence clinical practice. For instance, in some countries, healthcare professionals’ personal religiosity was closely tied to their willingness and ability to address patients’ spiritual concerns (34). Also, Mächler et al. (2023) explored the interrelationship between healthcare professionals’ personal spirituality, their spiritual competence, and attitudes toward addressing spirituality in clinical practice. Their study highlighted that a strong correlation exists between personal spirituality and the ability to perceive patients’ spiritual needs (35).

### Limitations

Despite the significant findings, it is essential to consider the limitations of this study. Firstly, the data were obtained from a specific sample of psychosomatic inpatients, which may limit the generalizability of the findings to other populations or outpatient settings. Secondly, the study employed a correlational design, making it challenging to establish causation between spirituality and treatment outcomes. It is possible that other factors not assessed in this study may be influencing the observed associations. Additionally, self-report measures, such as the TPV and TSI, are subject to biases and may be influenced by the patients’ individual beliefs and perspectives. Furthermore, the spiritual aspects investigated in this study were broad and multidimensional, which makes it difficult to capture the complexity of spirituality comprehensively. To build upon these findings, future research could utilize longitudinal designs and intervention studies to examine the causal relationships between spirituality and treatment outcomes. Moreover, employing diverse samples from different cultural backgrounds and spiritual orientations may provide a more comprehensive understanding of the role of spirituality in psychosomatic treatment.

## Conclusion

Despite correlations between psychosomatic health and spirituality as measured with TPV and TSI were rather small several spirituality items could be identified which seem to have a prominent connection to the diagnosis and development of psychosomatic health. Specific spiritual attitudes, such as access to inner stillness and connection to a greater whole, were linked to positive treatment responses and reduced symptom burden. These findings suggest potential benefits in incorporating spirituality into psychosomatic treatments, though its complex and multifaceted nature warrants further investigation.

## Data Availability

The raw data supporting the conclusions of this article will be made available by the authors, without undue reservation.
